# Advances in Waterborne Polyurethane and Polyurethane-Urea Dispersions and Their Eco-friendly Derivatives: A Review

**DOI:** 10.3390/polym13030409

**Published:** 2021-01-27

**Authors:** Arantzazu Santamaria-Echart, Isabel Fernandes, Filomena Barreiro, Maria Angeles Corcuera, Arantxa Eceiza

**Affiliations:** 1 Centro de Investigação de Montanha (CIMO), Instituto Politécnico de Bragança, Campus de Santa Apolónia, 5300-253 Bragança, Portugal; ipmf@ipb.pt (I.F.); barreiro@ipb.pt (F.B.); 2 Group ‘Materials + Technologies’, Department of Chemical and Environmental Engineering, Faculty of Engineering of Gipuzkoa, University of the Basque Country, Pza Europa 1, 20018 Donostia-San Sebastian, Spain; marian.corcuera@ehu.eus (M.A.C.); arantxa.eceiza@ehu.eus (A.E.)

**Keywords:** polyurethane and polyurethane-urea dispersions, synthesis methods, materials’ properties, alternative internal emulsifiers, sustainable strategies, renewable additives, processing methods, applications, legislation

## Abstract

Polyurethanes and polyurethane-ureas, particularly their water-based dispersions, have gained relevance as an extremely versatile area based on environmentally friendly approaches. The evolution of their synthesis methods, and the nature of the reactants (or compounds involved in the process) towards increasingly sustainable pathways, has positioned these dispersions as a relevant and essential product for diverse application frameworks. Therefore, in this work, it is intended to show the progress in the field of polyurethane and polyurethane-urea dispersions over decades, since their initial synthesis approaches. Thus, the review covers from the basic concepts of polyurethane chemistry to the evolution of the dispersion’s preparation strategies. Moreover, an analysis of the recent trends of using renewable reactants and enhanced green strategies, including the current legislation, directed to limit the toxicity and potentiate the sustainability of dispersions, is described. The review also highlights the strengths of the dispersions added with diverse renewable additives, namely, cellulose, starch or chitosan, providing some noteworthy results. Similarly, dispersion’s potential to be processed by diverse methods is shown, evidencing, with different examples, their suitability in a variety of scenarios, outstanding their versatility even for high requirement applications.

## 1. Introduction to WBPU and WBPUU Dispersions

Polyurethanes and polyurethane-ureas are a versatile family of polymers finding use in a wide range of applications such as biomedical, textile, automotive, paintings, adhesives, coatings, among others [1,2,3]. They can present various functionalities in their backbone, with the urethane as the main one (-NHCOO-). This group is formed by the reaction between an isocyanate (-NCO) and a hydroxyl group (-OH). In the case of polyurethane-ureas, besides urethane groups, urea groups (-NHCONH-) are also formed, namely by the reaction of isocyanates with amine groups (-NH_2_).

**Figure 1:**
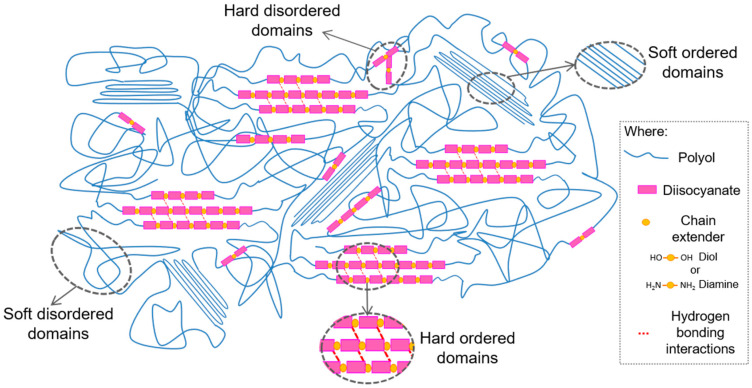
Scheme of segment conformations and hydrogen bonding interactions of polyurethanes or polyurethane-ureas, and the resulting ordered and disordered microdomains.

Among the distinct classes, segmented polyurethanes are block copolymers. They are composed of alternating blocks, namely the soft segment (SS), which consists of the polyol, and the hard segment (HS), formed by the isocyanate and the low molecular weight chain extender, a diol in the case of polyurethanes and a diamine in the case of polyurethane-ureas. These segments are usually thermodynamically incompatible, leading to phase separation, and thus to a microdomain structure which, depending on the chemical composition, can give rise to materials with a broad range of properties [4,5]. In general, the soft segment provides flexibility to the system, whereas the hard segment confers stiffness. Attending to the reactive mixture composition and reactants nature, soft and/or hard segments can be arranged in either amorphous disordered or crystalline ordered domains, as schematically shown in Figure 1, where segment conformations, hydrogen bonding interactions, and the resulting ordered and disordered microdomains is exemplified.

One of the most remarkable differences between urethane and urea groups is their capacity to form hydrogen bonding. In the case of urea groups, as both hydrogens could simultaneously participate in hydrogen bonding interactions, polyurethane-urea systems are characterized by stronger interactions, generally resulting in stiffer materials [6]. Conventional polyurethane and polyurethane-ureas, due to their hydrophobicity, give rise to solvent-borne systems, e.g., coatings and adhesives.

In the last years, environmental awareness intensification has led to diverse routes directed to the development of novel systems based on green chemistry approaches. This concept involves the implementation of pollution prevention policies to the design and development of new products [7]. In this context, some strategies including non-isocyanate synthesis routes, which avoid the use of isocyanate compounds and their implied restrictions [8], by the formation of polyhydroxyurethanes through amine and cyclic carbonate compounds reaction, are being implemented [9]. Besides, the replacement of petrochemical raw materials by naturally-based reactants, including biobased isocyanates [10], oils [9] or polysaccharides [11], are attractive vias for the synthesis of biobased polyurethanes or polyurethane-urea systems. Nevertheless, it should be highlighted that one of the most challenging goals deals with the replacement of the conventional solvent-borne systems to reduce volatile organic compounds emissions [12], positioning the development and use of waterborne products, namely waterborne polyurethane (WBPU) and waterborne polyurethane-urea (WBPUU) systems, at the forefront of polyurethane and polyurethane-urea based commodities [13]. The water compatibility character of these products can be achieved by adding an internal emulsifier [14], avoiding the use of organic solvents, thus reducing the generation of volatile organic emissions. Apart from environmental advantages, WBPU and WBPUU dispersions combine other excellent features: they exhibit high solids content and molecular weight, possess low viscosity and non-flammability properties and have good film-forming ability at room temperature [15,16]. Besides, films with properties similar to conventional polyurethanes can be obtained, such as excellent chemical resistance, high flexibility, adhesion to many polymers and surfaces, among others [17]. Depending on the reactants used, chemical structure, molar ratio, applied synthesis procedure, and internal emulsifier nature, WBPU and WBPUU dispersions with different final properties can be synthesized.

The internal emulsifier forms part of the polymeric chain, being covalently incorporated in the backbone of the polymer [14], providing stability to the formed nanoparticles during the phase inversion step leading to the dispersion formation. During this step, the hydrophobic moieties get arranged inside the particles forming the core. The hydrophilic ones, formed by the urethane and urea groups, which incorporate the emulsifier, get positioned at the particle surface (shell). Internal emulsifiers of different nature can be used to prepare dispersions, i.e., nonionic or ionic type, where the latter comprise cationic and anionic reactants [18].

The emulsifier content used must be at a minimum level to get stable dispersions, a concentration that depends on emulsifier nature, reactants type, and hard/soft segments ratio [19,20,21]. For example, Nanda and Wicks [20] analyzed the influence of the internal emulsifier dimethylol propionic acid (DMPA) content in the stability of WBPU dispersions observing that, for a fixed chemical composition, the required minimum DMPA content varied from 2 to 4 wt.%.

The effect of other parameters, namely polyols’ structure and molecular weight, are also known to impact dispersion particle size and viscosity, as evidenced by Haiyun Wang and co-workers [22]. In general, the particle size of the dispersions varies depending on the nature of the polyurethane chain, i.e., a higher hydrophilic character favors the obtainment of lower particle size dispersions, which result in higher viscosity products. Namely, the synthesis of high solids content polyester-based WBPUs presents less difficulties than polyether polyol counterparts due to ester groups’ capacity to form hydrogen bonds with water. However, the use of polyether-based systems can favor specific properties; e.g., the use of poly(tetramethylene ether) glycol (PTMG) facilitates water vapor permeability in coated fabric applications.

## 2. Internal Emulsifiers in WBPU and WBPUU Dispersions

There are different types of internal emulsifiers for the synthesis of WBPU and WBPUU dispersions. Attending to their nature, emulsifiers can be classified into nonionic or ionic, where the ionic ones can also be subdivided into cationic or anionic, depending on the charge of the functional groups conferring hydrophilicity and stability to the system [23].

**Figure 2:**
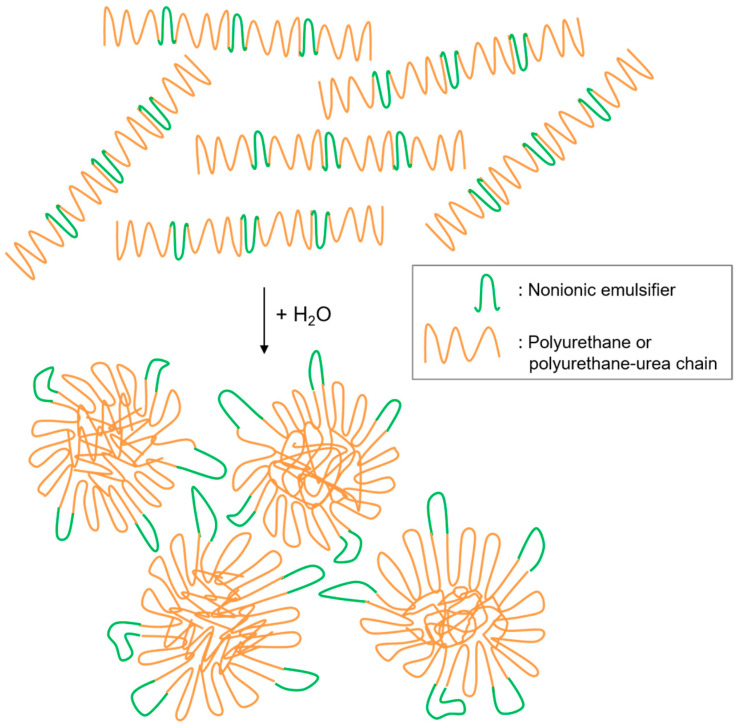
Scheme of particle’s formation in nonionic WBPU or WBPUU dispersions.

The stabilization mechanism of particles in nonionic dispersions is based on a steric stabilization mechanism usually provided by hydrophilic soft segments, as shown in Figure 2. The portions of the chains containing the nonionic segments spread out to the continuous phase, that is, to the water phase, hindering the coalescence effect among the formed polymeric nanoparticles [24].

**Figure 3:**
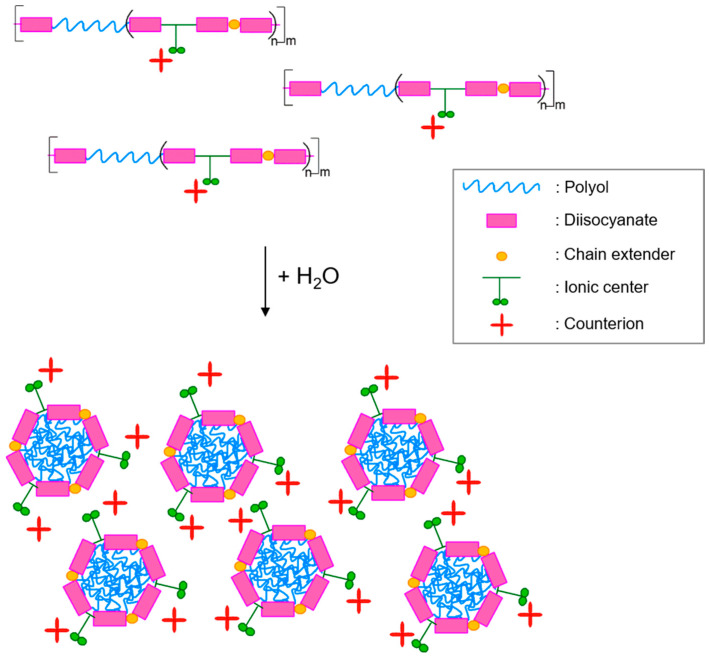
Scheme of particle’s formation in ionic WBPU or WBPUU dispersions.

Regarding ionic emulsifiers, anionic and cationic WBPU or WBPUU differ in the ionic center pendant from the polymer backbone. In the case of the anionic emulsifiers type, carboxylic or sulfonated acids are usually used. In the cationic systems, tertiary amines are generally incorporated, these last ones needing to be neutralized (in anionic systems) or quaternized (in cationic systems) to form the corresponding salts [1,17]. In ionic WBPU and WBPUU dispersions, an electrostatic stabilization takes place, based on the electrical double layer mechanism, by adding a counterion to the emulsifier group previously to the dispersion step. The counterion is the ion present in the system responsible for maintaining the ionic center’s electric neutrality [25]. Figure 3 shows the particle’s formation mechanism of ionic WBPU and WBPUU dispersions.

When water is added to the polymer, the ionic groups will become placed at the surface of the particles surrounded by the counterions, forming the electrical double layer [26,27] and constituting the particle’s shell. The hydrophobic domains will be agglomerated in the inner part of particles making their core [18]. Thereby, the electrical double layer’s interference among particles results in their mutual repulsion, leading to dispersion stabilization through a mechanism based on the repulsive electrostatic interactions [1,28].

## 3. WBPU and WBPUU Synthesis Methods

### 3.1. Overview of The Most Used Synthesis Procedures and Their Characteristics

The WBPU and WBPUU production methods progressed over the years to increasingly sustainable synthesis routes. The initial method, namely the acetone method, evolved into designing an alternative method, the prepolymer method, aiming to elude the restrictions of the patent held by Bayer (e.g., first development on polyurethane dispersions started in the 1960s, and later in the 1980 the continuous production of dispersions was implemented) [29,30]. Nevertheless, the use of N-methyl-2-pyrrolidone (NMP), a highly toxic solvent widely used to dissolve the hydrophilizing agent (e.g., DMPA) in the prepolymer method, required the modification of the process to avoid legislation limitations regarding the NMP. In this context, the modified prepolymer method displaced the conventional prepolymer method, leading to NMP-free dispersions. Even with “green” connotations, since solvent-free products are achieved, these processes present the disadvantage of needing organic solvents during the synthesis. Thus, current methods focus on developing green products through sustainable approaches, i.e., without using organic solvents during the synthesis. Considering the diversity of available synthesis methods and the wide range of chemical compositions, Table 1 summarizes these variables’ influence on different WBPU and WBPUU dispersion systems’ final properties, as gathered from the literature.

### 3.2. Traditional Synthesis Methods

#### 3.2.1. Acetone Process

The acetone process formerly patented by Bayer uses a hydrophilizing diamine (N-(2-aminoethyl)-2-aminoethanesulfonic acid) having the double role of chain extender and internal emulsifier, thus leading to WBPUU dispersions. Briefly, the process starts with the synthesis of a prepolymer in bulk trough polyol and isocyanate reaction, followed by the addition of acetone to control viscosity and solubilize the N-(2-aminoethyl)-2-aminoethanesulfonic acid, thus conducting to a chain extension step in a homogeneous medium, that is, prior to the phase inversion step. The inversion phase is then conducted by the addition of water under vigorous stirring to the synthesized polymer in acetone. The final step comprises the extraction of the organic solvent. The acetone process enables controlling the molecular weight, and particle size and distribution with high reproducibility, considering that the polymer synthesis is carried out in a homogeneous solution [20,36]. In fact, the acetone dilution avoids viscosity constraints, being recovered to be reintroduced in the system. Nevertheless, the used high quantities of acetone and its purification process could be considered the main drawback of the process [46].

#### 3.2.2. Prepolymer and Modified Prepolymer Processes

Traditionally, the prepolymer process, designed as an alternative to the patented acetone process, is carried out by synthesizing an NCO-terminated prepolymer of moderate molecular weight (around 8000 g mol^−1^) [39] comprising the reaction of an isocyanate, a polyol, and an internal emulsifier (usually DMPA). Facing to the importance of achieving high molecular weight polymers due to the need for high performance properties in service [47], the increase of the molecular weight is later conducted through a chain extension step. Considering the insolubility of the internal emulsifier (in the prepolymer and acetone), a highly polar co-solvent (e.g., NMP) whose high boiling point hinders its removal from the final product, is needed. Before the dispersion step, the ionic groups of the internal emulsifier are neutralized using a tertiary amine, and the viscosity of the medium can be adjusted with acetone. The prepolymer is then dispersed in water, and (afterward or during the addition of water) the molecular weight of the final polymer is increased through a chain extension reaction with di- or polyamines in a heterogeneous medium (e.g., water and acetone medium). It should be worth noting that by this approach, even it is less widespread, the chain extension step can also be carried out before the phase inversion step (using a diol or diamine), leading in this case to a homogeneous chain extension step strategy. Analogously to the acetone process, the incorporated acetone can be removed in a final step [39] even though the NMP co-solvent will remain in the dispersion.

**Figure 4:**
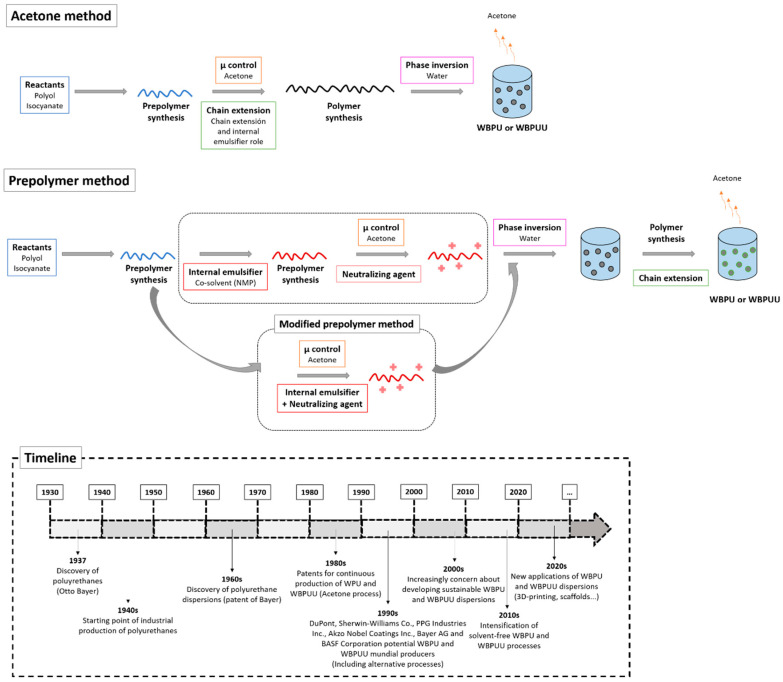
Scheme of the main steps of acetone and prepolymer methods for the preparation of WBPU and WBPUU and the progress timeline in the field of WBPU and WBPUU.

To avoid the hazard problems derived from NMP containing dispersions, the process evolved into the modified prepolymer method. This approach involves a slight variation in the conventional prepolymer method availing the possibility of synthesizing NMP free WBPU and WBPUU systems. The combined incorporation of the internal emulsifier with the neutralizing agent enables its dissolution in acetone, avoiding NMP as co-solvent. Furthermore, compared with the acetone process, the modified prepolymer method ensures the preparation of WBPU and WBPUU using lower quantities of acetone to control the viscosity considering the lower molecular weight of the prepolymer before the inversion step. In Figure 4, a schematic representation of the principal steps of both acetone and prepolymer methods is presented, including the progress timeline in the field of WBPU and WBPUU.

### 3.3. Alternative Solvent-Free Methods

Research investment in the field of environmentally-friendly materials is now focusing on alternative synthetic routes to achieve not only solvent-free products, but also entirely solvent-free synthesis processes. For example, Wang and co-workers [45] designed a solvent-free WBPU synthesis route through a strategy using a dispersing stage at elevated-temperature (avoiding the need of organic solvents). State of the art emphasizes the importance of carrying out the dispersion step at low temperatures (room temperature) to prevent the NCO groups’ side reactions with water and thus the formation of materials with poor properties. By contrast, in this work, based on the principle that high temperatures favor low viscosities, after prepolymer synthesis, a small amount of water is added at high temperature to pre-disperse the prepolymer, thus reducing the viscosity to proceed with the prepolymer dispersion at low temperature. By this strategy, the cooling and solvent-removing steps characteristic of the traditional methods are avoided shortening the synthesis process’s duration and reducing energy consumption.

Nevertheless, considering the impossibility of avoiding secondary reactions completely (between NCO and water), this parameter needs to be controlled to reach an acceptable value, namely a NCO retention > 90%, for not compromising the mechanical properties of the materials. In another work, Xiao et al. [43] synthesized WBPUU dispersions by a solvent-free strategy based on a hydrophilic chain extender, sodium 2,4-diaminobenzenesulfonate (SDBS). Water was employed as the dissolving agent of the solid reactants and to control viscosity, enabling the polymer’s dispersibility without adding organic solvents. The formed products have shown similar properties (e.g., mechanical and water resistance) to conventional WBPU or WBPUU dispersions. Similarly, Yong and co-workers [44] synthesized solvent-free WBPU mats using DMPA and sodium 2-[(2-aminoethyl)amino] ethane sulfonate (SAAS) as the chain extender agents, without using organic solvents in the process. The large excess of isocyanate in the medium during the pre-polymer step favored its dual action as the reagent and the solvent medium for the polymerization process.

## 4. A Step beyond Conventional Internal Emulsifiers

Conventionally, WBPU, and WBPUU are prepared according to nonionic or ionic dispersing strategies. The nonionic WBPU and WBPUU dispersions, which are based on the use of hydrophilic internal emulsifiers, e.g., polyethylene oxide, or lateral/terminal ether moieties [17], were not so widespread as the ionic counterparts, partly due to the weaker hydrophilic character of the nonionic emulsifiers, which difficult the dispersion in water. Nevertheless, due to their lower toxicity, better electrolyte stability, and resistance to shearing at low temperature, nonionic dispersions are the most suitable WBPU and WBPUU formulations for breathable coating applications, dyeing, finishing, and cosmetics, among others [23,48,49]. The ionic (anionic and cationic) dispersions are more widespread and extensively used in diverse applications, including adhesives, textiles, coatings, automotive topcoats or packaging films [50]. Nevertheless, it has to be worth noting that the high level of nitrogen in cationic-based dispersions, implies their tendency for yellowing. This fact implies constraints for some applications, and thus cationic dispersions are not so widespread compared to the industrial applications of anionic dispersions [18,23]. Among anionic emulsifiers, particularly DMPA [51,52,53], 2,2-bis(hydroxymethyl) butyric acid (DMBA) [54,55], and sulfonated agents [56,57] are commonly employed in the synthesis of WBPU and WBPUU [58]. Concerning cationic emulsifiers, N-methyldiethanolamine (MDEA) is the most used one [58,59,60].

The progressive restrictions in the actual legislation boosted the synthesis methods’ evolution to more sustainable pathways. Some approaches comprise the design of novel dispersions with alternative emulsification strategies, covering the mixture of conventional emulsifiers, namely the combination of nonionic and ionic agents, novel biobased hydrophilic chain extender emulsifiers, or hydrophilizing biobased polyols, among others. In Table 2, some examples of WBPU and WBPUU dispersions based on these evolving concepts, i.e., from using conventional to alternative emulsifier agents, are summarized.

The development of WBPU and WBPUU dispersions containing both types of internal emulsifiers, i.e., nonionic and ionic types simultaneously, has gained attention to obtain films with improved and balanced properties by combining the beneficial advantages provided by each emulsifier. For example, Yen and co-workers [23] synthesized cationic-nonionic WBPU by incorporating poly(ethylene oxide) (PEO) as side-chains acting as the nonionic moiety, and MDEA as the cationic one. Considering that the addition of PEO into the polymeric chains could improve specific parameters such as the hydrophilic character, the conductivity, and phase-mixing of soft and hard segments, these materials were tested for fabric coating applications. Therefore, cationic-nonionic polyurethanes with PEO side-chains of different lengths were prepared. The application to coat nylon-based fabrics was investigated in terms of waterproof capacity, antiyellowing effect, and water vapor permeability properties, a strategy that revealed promising results. Li and co-workers [84] developed another cationic-nonionic WBPU product using a synthesized nonionic segment (polyoxyethylene alkyl amine, PAE) containing poly(ethylene glycol) (PEG) and MDEA (cationic emulsifier), following a specifically designed reaction sequence to locate the cationic groups in the SS phase, and have analyzed the effect of chain terminal ions in the WBPU final properties. They found that the used strategy can tailor the particle size of the dispersions, promoting their efficient packing, which was a decisive parameter in achieving high solid content products while holding low viscosity values. In another work, Lijie et al. [85] have developed anionic-nonionic WBPU dispersions based on the principle that the critical micelle concentration, and the surface tension, can be reduced by using ionic and nonionic surfactants, whose combination improves their efficiency [86]. This assumption was the basis for the synthesis of stable WBPU, characterized by high solids content and water resistance but requiring low hydrophilic monomers concentrations. In this work, nonionic PEG of different molecular weights and anionic DMPA were combined through different synthesis schemes to prepare several WBPUs, being observed that the suitable combination of both emulsifier components, even at low contents, resulted in stable dispersions. Anionic groups promote the formation of the electrical double layers contributing to the electrostatic repulsion effect among particles [87], whereas nonionic hydrophilic segments could reduce the interfacial tension and thus facilitate the dispersion formation.

**Figure 5:**
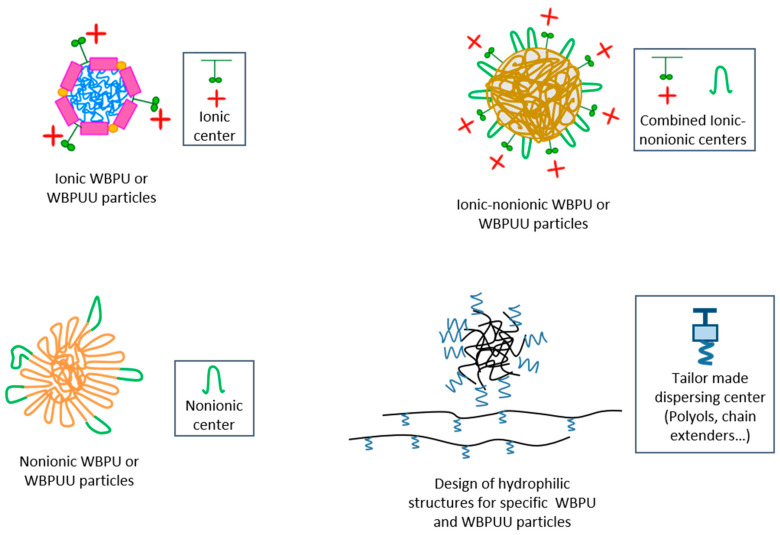
WBPU or WBPUU particles’ architecture based on their hydrophilic center.

The importance of using biobased reactants in green synthesis routes of WBPU and WBPUU dispersions has also driven the attention to the field of emulsifier agents. For example, Bahadur and co-workers [68] developed a biodegradable elastomeric WPUU dispersion using lysine holding a double function, i.e., as the internal emulsifier and the chain extender, avoiding the use of petrochemical-derived internal emulsifier reactants. With the same purpose, but following an alternative approach, Kumar and Palanisamy [69,70] developed a vegetable oil-based polyol characterized by presenting hydroxyl and carboxyl groups in the triglyceride backbone for further use in the preparation of anionic WBPU and polyurethane-imide dispersions, conforming a DMPA-free synthesis strategy. For this purpose, a synthetic esterification route was designed by modifying the hydroxylated groups of cottonseed oil with maleic anhydride. Additionally, Omran and co-workers [64] synthesized an alternative carboxyl containing polyol prepared by the methoxylation and saponification of sunflower oil, which was later used to synthesize WBPU dispersions. Aiming to summarize the diverse type of structures that can be designed in the development of WBPU and WBPUU particles, Figure 5 includes a scheme of the most representative architectures based on the described approaches.

## 5. WBPU and WBPUU Added with Renewable Additives

Despite the great variety of specifications offered by WBPU and WBPUU based products, e.g., composites, the enhancement of some specific properties is an important strategy having in view certain applications. Based on this fact, the incorporation of renewable additives, like water-dispersible nanoentities, which contribute to reinforce the environmentally-friendly character of WBPU and WBPUU dispersions, is presented as a suitable opportunity to face this challenge. Therefore, a brief overview of different examples will be performed in the next sections, focusing on the available nanoreinforcements, incorporation routes, and processing methods. Moreover, the capability to modulate the final properties of the materials through these strategies will be highlighted.

### 5.1. WBPU and WBPUU Added with Nanocellulose

**Figure 6:**
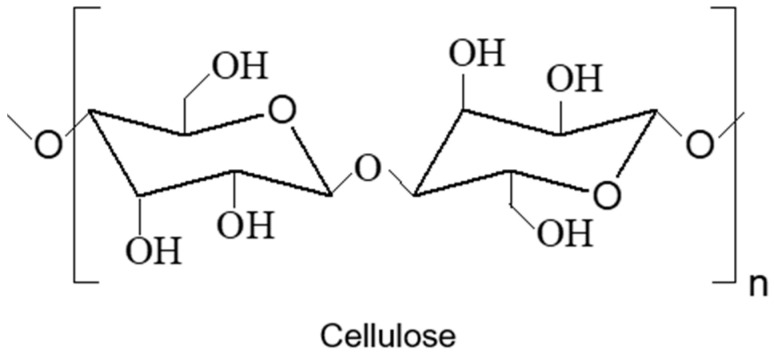
Chemical structure of cellulose.

WBPU and WBPUU dispersions’ hydrophilic character facilitates the addition of water-dispersible nanoentities, promoting their incorporation through simple routes. Among others, nanocelluloses, in the form of nanofibers (CNF), and nanocrystals (CNC), also known as nanowhiskers (CNW), constitute attractive reinforcements bringing to the materials where they have been incorporated biodegradability, recyclability, renewability, and biocompatibility [88]. Their high specific modulus (modulus/density) makes them attractive to provide enhanced mechanical properties to the reinforced composites [89]. Nanocelluloses are formed by cellulose, a renewable polymer composed of D-glucose units linked by β-(1,4) glycosidic bonds, as shown in Figure 6 assembled in hierarchical structures by hydrogen bonding interactions.

Nanocellulose, both CNF and CNC, was used in several works, including the incorporation into WBPU and WBPUU, generally resulting in the enhancement of the mechanical behavior in terms of modulus and stress values. Namely, the effective CNF addition to a WBPU (10 wt.% of CNF) favored the increase of the tensile strength up to 61% compared to the neat WBPU [90]. In the case of CNC incorporation into a WBPU matrix, an amount of just 1 wt.% of this nanoreinforcement was able to improve the tensile strength value up to 125%, doubling the modulus value of the matrix [91].

Different strategies can be employed regarding the nanoreinforcement incorporation route to achieve an effective dispersion of the nanocellulose into the WBPU or WBPUU. For example, Santamaria-Echart et al. [92] analyzed two strategies for the addition of cellulose nanocrystals (CNC) to a WBPU. These include the addition and homogenization by sonication after WBPU synthesis, and in situ routes where the CNC were incorporated during the synthesis process dispersed in the water phase used in the dispersion step. The morphological analysis showed that the CNC assumed different dispositions in the matrix, being partially embedded in the WBPU nanoparticles in the case of in situ strategy, thus leading to different thermal, mechanical, and hydrophilic behavior. de Oliveira Patricio et al. [93] also analyzed different incorporation routes of CNC to a WBPU, namely during the dispersion step after prepolymer synthesis, or mixed in the polyol in the prepolymer synthesis step. They concluded that the hydrogen bonding ability between the CNC and the WBPU was influenced according to the step where they were incorporated. The established levels of interactions can control the morphology and the interfacial adhesion that define the mechanical properties.

In terms of processability, Li et al. [94] prepared mats using an alternative process to film casting. The process comprised filtering a bleached eucalyptus Kraft pulp slurry into a cupreous mesh followed by immersion into a WBPU dispersion, and then by drying. Final composites with improved thermomechanical properties were obtained.

Guo-Min et al. [95] prepared thermoset nanocomposites based on a two-component waterborne polyurethane (2K-WBPU) and CNC. The addition of CNC promoted their interaction with the 2K-WBPU matrix, forming a rigid nano-phase acting as crosslinking points in the polymer matrix, enhancing mechanical properties and thermomechanical stability of the nanocomposites. Zhao et al. [96] showed that adding just 1 wt.% of CNC to a WBPU could improve the tensile strength up to 15%. Furthermore, the nanocomposites’ anti-felting effect in wool samples showed an area-shrinking reduction from 5.24 to 0.70%, values determined using a machine-washable technical standard, thus corroborating their great potential in the field of textile applications. Therefore, nanocellulose becomes an attractive renewable nanoreinforcement for the preparation of WBPU composites.

### 5.2. WBPU and WBPUU Added with Starch

**Figure 7:**
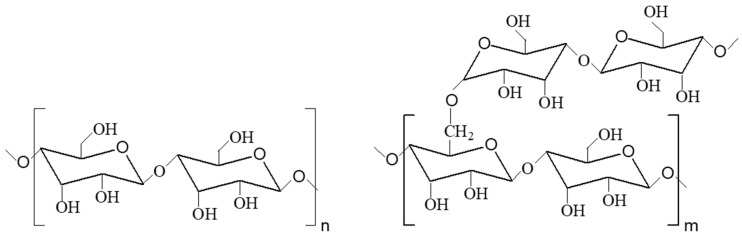
Chemical structure of amylose and amylopectin.

Starch (St), a natural hydrophilic polymer composed of amylose and amylopectin (Figure 7), is an alternative to cellulose. Starch results in an appealing option in terms of renewability, versatility, availability, and low cost, mainly due to its biodegradability [97], which is of high interest for applications that need to fulfill biodegradation requirements.

In this context, starch was used to provide, or enhance, those particular properties in the WBPU and WBPUU, configuring an attractive eco-friendly alternative. For example, Lee and Kim [98] synthesized a WBPU incorporating vinyltrimethoxysilane (VTMS) modified starch covalently bonded to the polyurethane chains. They observed that the miscibility of both components and the biodegradability of the material, which was compared with a just blended WBPU-starch system, was improved. With this purpose, after obtaining the WBPU dispersion, the VTMS modified starch was incorporated, and the mixture homogenized for the future addition of a photoinitiator. The resultant mixture was cast and cured by UV radiation, resulting in a multifunctional crosslink network structure. The effect of St in the biodegradability process was extensively analyzed using an amylase solution (enzyme capable of catalyzing the breakdown of starch structure into sugars) in a buffer solution. Results showed that when using the amylase medium, the samples’ weight loss was higher than the starch amount presented in the system, implying that the formed network containing St induced the degradation of the polyurethane chains. In the case of WBPU-starch blends, the weight loss was equivalent to the blended St, indicating that it was degraded without promoting the WBPU matrix’s biodegradation. Travinskaya et al. [99] also incorporated St into a WBPU dispersion during the synthesis process (WBPU/starch). The prepolymer dissolved in acetone was dispersed in a water phase containing starch, resulting in the combination of the chain extension and dispersion stages. An alternative strategy was also designed by preparing mechanical mixtures (WBPU + starch) for comparison purposes. In this case, the starch in the form of an aqueous solution, was added to the already synthesized WBPU dispersion. It was observed that the WBPU/starch method ensured the chemical and physical interaction between the WBPU matrix and the starch, enhancing the stability to aggregations and the film-forming ability, whereas, in the mechanical mixtures, the intermolecular interactions of each component prevailed. Regarding the film’s biodegradability, the adhesion capacity of microorganisms (*Bacillus subtilis*) to the film’s surface was evaluated. Results showed that samples containing starch incorporated during the synthesis process presented higher biodegradability than the matrix alone. This fact implied that these samples biodegraded as an entire system, unlike the ones prepared by the mechanical mixture. Furthermore, WBPU/starch samples resulted in systems more susceptible to alkaline and acid hydrolysis than the base WBPU matrix. Thus, the work showed the possibility of synthesizing degradable WBPU based on renewable starch polysaccharides.

Apart from biodegradability, starch-based systems can also enhance other properties such as the mechanical behavior of films, as evidenced by Zou et al. [100] in a representative work where, by acid hydrolysis, starch nanocrystals with a distinct platelet-like (similar to exfoliated silicate) were isolated and incorporated into a WBPU. These specific nanocrystals morphology allowed their addition up to 30 wt.% resulting in an enhancement of the Young’s modulus around 67-fold compared with the WBPU matrix. When 10 wt.% of starch nanocrystals were added, the most significant enhancement in the strength and Young’s modulus, in comparison with the base matrix, was 1.8 and 35.7, respectively. This effect was due to the enduring and effective stress transfer observed in the nanocomposites, attributable to the strong starch nanocrystals-WBPU interactions.

### 5.3. WBPU and WBPUU Added with Chitosan

**Figure 8:**
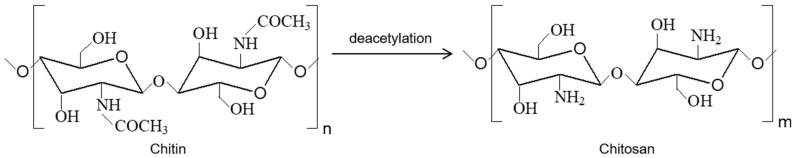
Deacetylation of chitin to chitosan.

Chitosan (Cht), a chitin derivative, is a biopolymer characterized by presenting non-toxicity, biocompatibility, biodegradability, and low cost [101]. To obtain chitosan, chitin, a polysaccharide present mainly in the exoskeleton of crustaceans and other invertebrates, is subjected to a deacetylation treatment (Figure 8). When a deacetylation degree (DD) of around 50% is reached the product is considered as chitosan, a polymer that is soluble in acidic media [102].

Due to its characteristics, chitosan is widely employed in materials focusing on antimicrobial applications and in medicine. Thus, chitosan incorporation into WBPU and WBPUU dispersions mainly focus their application in these research areas. El-Sayed et al. [103] synthesized WBPU using chitosan of different molecular weights as chain extenders. With this purpose, the chain extension step was carried out during the phase inversion step by adding dropwise a chitosan solution prepared using 1% of acetic acid. Then, acrylic fabrics were treated with the prepared dispersions and analyzed concerning their antimicrobial properties envisaging their use as blankets or carpets in hospitals. Results showed the effective antimicrobial activity of the fabrics, which remained unchanged after 15 washing cycles. Bankoti et al. [104] prepared hydrogels by blending WBPU and chitosan to obtain dried scaffolds, which were later crosslinked using a sodium tripolyphosphate solution. The WBPU/Cht blend becomes self-organized in a macroporous structure after the drying stage, facilitating protein adsorption and improving scaffolds’ stability in aqueous enzymatic environments, which make viable their use in biological media. The hydrogels presented good cytocompatibility, hemocompatibility, and, in the case of some specific compositions, pointed out for effective biocompatibility when tested as biomaterials for wound healing applications.

Considering the significant variability of the entities used as reinforcements or additives, their preparation method, and the incorporation route for their addition, several factors can influence the generated materials’ final behavior. To get some insights into this variability, Table 3 summarizes different approaches used in the preparation of the reinforcements, subsequent incorporation route in the WBPU and WBPUU, and their derived composites and blends.

## 6. Processing Methods and Applications of WBPU and WBPUU

It is well recognized the easy film-forming ability of WBPU and WBPUU dispersions by the conventional casting method. Nevertheless, their versatility provides the opportunity to use alternative processing techniques, namely electrospinning or 3-D printing, conferring to dispersions the potential to be employed in advanced applications aside from the common uses as adhesives paintings or coatings. Among others, the possibility of processing 3-D interconnected porous scaffolds by freeze-drying is an attractive strategy to prepare materials with adapted properties. Tailoring the porous diameters can provide a way to modulate the adhesion and proliferation of human cells focused on tissue-engineering applications [113]. The freeze-drying method can also be employed to prepare hydrogels, as showed by Wang et al. [114]. After synthesizing the WBPU dispersion, mixtures with poly(vinyl alcohol) (PVA) were prepared and freeze-dried to obtain WBPU/PVA composite hydrogels, which were extensively analyzed, outlining their applicability in the field of wound dressings in medical devices.

The use of PVA, or other similar polymers such as PEO, facilitates the spinnability of WBPU dispersions by electrospinning technique allowing the preparation of mats. These polymers act as polymer templates enabling nanofibers’ formation with subsequent PEO extraction if needed, resulting in WBPU mats [115]. Yang et al. [116] prepared WBPU/PVA mats observing that the polymer concentration and weight ratios influenced the electrospinnability and morphology of the electrospun mats, which presented, in general, high water-uptake values, having potential for wound dressing applications. Other electrospun WBPU/PVA mats were prepared by Wu et al. [117]. In this case, modulation of nanofiber diameters by changing the WBPU/PVA ratio was attempted, giving rise to interconnected porous structures, similar to those found in natural extracellular matrices. Biodegradability, non-toxicity, and biocompatibility of the electrospun mats were also improved, facilitating the attachment and proliferation of cells, making them attractive biomaterials for natural tissue repair applications. It is worth noting that this processing method also allows the incorporation of nanoentities, and thus of nanocomposites preparation. For example, in previous works developed by Santamaria-Echart et al. [118], mats with incorporated cellulose nanocrystals were prepared by electrospinning, being observed that their content and incorporation route could tailor the morphology of the fibers and surface properties of the mats. To put in evidence the wide range of processing methods (after their synthesis) that can be applied with WBPU and WBPUU, and application fields, a set of examples have been summarized in Table 4.

## 7. Current Regulation of WBPU and WBPUU Dispersions

In the last twenty years, the European Union has made significant efforts to promote the use of safe and sustainable chemicals, increasing the protection of human health and the environment against hazardous chemicals. Thus, the European Commission has adopted a chemical strategy for sustainability as a starting point towards a toxic-free environment. This strategy arises from the carried out European policy of the last two decades, in what concerns the chemicals regulation. Since 2004 several Regulations and Directives have imposed restrictions on a wide array of conventional chemical products used in various industrial processes and products, leading the industry and the scientific community to search and develop new friendly alternatives. In this context, the WBPU and the WBPUU materials appeared to be interesting and viable alternatives, once they can be produced by green processes and contain low amounts, or even be exempt, of organic solvents, beyond having custom-made properties, which make them one of the most versatile polymeric materials [46]. Despite this, the used chemical systems and processes have also been limited by the European Regulations. Firstly, the Registration, Evaluation, Authorization, and Restriction of Chemicals (REACH) Regulation (EC) 1907/2006 [133], demanded the registration of all chemical substances, products, and mixtures of the European Union, European Regulation 1907/2006 [133]. After, based on the gathered information and the criteria of article 57 of the REACH Regulation, a candidate list of substances of very high concern (SVHCs) was elaborated, Commission Regulation 1272/2008, [134], which was periodically updated, in the last years, by the European Chemicals Agency (EChA). Among the listed substances, the ones classified as carcinogenic, mutagenic, and toxic for reproduction (CMTR) are divided into two categories, 1A and 1B, depending on the level of concern and the need for a special authorization from EChA to be imported, used or commercialized. The maximum content allowed in mixtures and final products was also established, following the intended final application.

Analyzing the components of the WBPU and WBPUU base chemical systems, several restrictions and limitations are identified from the perspective of the legal impositions. Components such as the diisocyanates, the di-substituted tin-based catalysts, some of the diamines used as chain extenders, or the co-solvent N-methyl-2-pyrrolidone, NMP, have been restricted due to their classification (Table 5). Presently, the diisocyanates are strongly limited due to their respiratory and cutaneous highly sensitizer character, leading to chronic diseases after prolonged exposition. Recently, from August 2023 onwards, it was established that diisocyanates would be only allowed, in final products or mixture, in its free form (unreacted) in contents lower or equal to 0.1% (*w*/*w*), according to the Commission Regulation 1149/2020 [135]. Additionally, the industrial or professionals must complete training on the safe handling of diisocyanates before their use. For the diamines-based chain extenders, hydrazine was classified as carcinogenic in 2011 by the Commission Regulation 1272/2008 [134], being its usage forbidden once there are other friendly equivalent alternatives as 1,6-hexamethylene diamine or ethylenediamine. However, ethylenediamine was also recently pre-registered on the SVHCs list due to its respiratory sensitizing properties, EChA Report in 2018, [136] being expected to increase, in a near future, the maximum allowed unreacted content.

Regarding the content of the co-solvent NMP, it is already limited to a maximum value of 5% (*w*/*w*) in final products since 2015 onwards, according to the Commission Regulation 1272/2008 [134], being recently lowered to 0.3% (*w*/*w*) due to the NMP high toxicity referred to the Commission Regulation 588/2018, [137]. From an overall analysis of the WBPU and WBPUU base chemical systems legal framework, an increase of the raw-materials restrictions is foreseen for the next years, implying a constant need for research and development of friendly alternatives or even synthesis processes adaptation. Nevertheless, it is important to mention that these restrictions are relative to the pure raw material components and their presence in mixtures, thus not valid for final products where they are fully reacted, meaning that these compounds can be used as raw materials after the proper REACH authorization.

Analyzing the European legal impositions from the final application field perspective, specific Regulations are applied. When considering the WBPU and WBPUU as coatings and paints, their application is ruled by the Directive 2004/42/CE [138], which imposes limits for volatile organic compounds emissions, which are established based on the product type, e.g., paints (30–750 g/L), and coatings (140–840 g/L). For indirect food contact, and according to the information summarized in Table 5, restrictions such as specific migration limits for internal emulsifiers and chain extenders are imposed by the Commission Regulation 10/2011 [139], which was updated by the recent Commission Regulation (EU) 1245/2020 [140]. In the case of applications where direct human contact is inherent, such as footwear, textile, or accessories, Regulation 1513/2018 [141] restricts the presence of NMP in the final product to 0.3% (*w*/*w*). Concerning WBPU and WBPUU materials in medical applications, more restrictive criteria are considered. Namely, this is reflected by the Regulation 745/2017 [142] on Medical Devices, which imposes excluding materials in medical applications based on the endocrine-disrupting chemicals and CMR, if safer alternatives are available. Within this scope, these substances can only be present in medical materials at contents equal or lower than 0.1% (*w*/*w*) if their use is justified by fulfilling all the specific criteria defined in Annex I of the regulation, which includes biocompatibility and cytotoxicity studies. It is worth to mention the actual struggle of the medical materials area on the replacement of PVC-based products due to the presence of additives classified as endocrine-disrupting chemicals. Within this context, the WBPU and WBPUU based materials are considered feasible and attractive alternatives due to their ecofriendly character associated with their higher performance technical properties.

## 8. Conclusions

This work presents a general overview of the WBPU and WBPUU dispersions covering from introductory chemistry concepts and synthesis methods to the current legislation and trends towards more sustainable systems. The progressive restrictions of the actual legislation to promote green chemistry and synthesis strategies are being addressed through diverse approaches, including totally solvent-free synthesis methods, and specific combinations or alternative internal emulsifiers. In this context, this review provides a global overview of the actual pathways. The possibility of adding renewable entities (nanocellulose, starch, and chitosan) to the WBPU and WBPUU dispersions was analyzed, highlighting the enhancement of the systems’ environmentally-friendly character while improving or conferring functional properties to the final products. Finally, the increased interest in WBPU and WBPUU dispersion processing techniques (electrospinning or 3-D printing) was also analyzed, considering the opportunity to broaden these systems’ applicability to advanced new products apart from their conventional uses in fields such as adhesives, paintings, or coatings. The versatility in composition, properties, processing and applicability of WBPU and WBPUU makes them promising materials that may evolve to new fields needing novel and specific requirements. To be highlighted, the evolution towards green systems, namely by the use of natural and biobased raw materials, as well as by the adoption of sustainable synthesis routes, including totally solvent-free processes. These strategies respond to high-service quality requirements such as mats for biomedical and tissue engineering applications, textile coatings or finishing agents, and membranes for pollution and purification approaches.

## Figures and Tables

**Table 1:** Effect of WBPU and WBPUU synthesis methods and composition on dispersion’s properties: chain extension medium (homogeneous or heterogeneous), solids content (SC), pH, zeta potential (Z_pot_), average particle size (PS) (Unimodal distribution (UmD), Bimodal distribution (BmD)), viscosity (µ), weight average molecular weight (M_w_), number average molecular weight (M_n_), and polydispersity index (PI).

Synthesis Method	Chemical Composition	Chain Extension Medium	SC (wt.%) pH Z_pot_ (mV)	PS (nm)	µ (cp)	M_w_ or M_n_ (g mol^−1^) PI	Ref
**Modified prepolymer**	PTMG/DMPA/IPDI/BD/DBTDL (reactants added simultaneously—one-pot reaction)	Homogeneous	SC: 20 - -	116 (UmD)	-	-	[31]
**Modified prepolymer**	PTMG/DMPA/IPDI/BD/DBTDL (reactants added step-by step—step-wise reaction)	Homogeneous	SC: 20 - -	50 (UmD)	-	-	[31]
**Modified prepolymer**	PPG/DMPA/IPDI/BD/DBTDL (reactants added simultaneously—one-pot reaction)	Homogeneous	SC: 50 - -	100–200 and 1500–2000 (BmD)	100-600	M_w_: 9800 PI: 1.85	[32]
**Modified prepolymer**	PPG/DMPA/IPDI/BD/DBTDL (reactants added step-by step—step-wise reaction)	Homogeneous	SC: 40 - -	PS: 200–700 (UmD)	100-1550	M_w_: 10,020 PI: 1.83	[32]
**Modified prepolymer**	PCHDO/PPG/PBA/DEG/DMPA/IPDI/HZ	Homogeneous	SC: 39 pH: 8.0–8.2 -	With PPG: 61 (UmD); with PBA: 67 (UmD); with PHDO: 73 and 247 (BmD)	-	-	[33]
**Modified prepolymer**	LO/DMPA/IPDI/HDI/HEMA/DBTDL	Homogeneous	SC: 36 pH: 9.14 -	261 and 5001 (BmD)	389	-	[34]
**Modified Prepolymer**	NPG/PCL/DMPA/AA/MDI/EDA/BHT/DBTDL	Homogeneous	SC: 30 - -	-	-	M_n_: 9600–16,200 PI: 1.6–2.4	[35]
**Modified prepolymer**	PPG (1000; 2000 g mol^−1^) /DMPA/IPDI/BD/HEMA/Irgacure 184/DBTDA	Homogeneous	SC: 30 - -	With PPG (1000): 26–44 (UmD); with PPG (2000): 66–103 (UmD)	-	-	[36]
**Modified prepolymer**	SFO/AA/IPDI/MDI/HDI/BD/DBTDL/DABCO/Ti (i-Pr)_4_	Homogeneous	SC: 27–30 - -	91–125 (UmD)	-	-	[37]
**Modified prepolymer**	PTMG/DMPA/H_12_MDI/APS/HEMA/IBOA/HDFDMA/BA/DBTDL	Homogeneous	- - -	48–122 (UmD)	22-39	-	[38]
**Modified prepolymer**	Desmophen 1019-55/DMPA/IPDI/BD/BOL/DBTDL	Homogeneous	SC: 25–45 - -	25–250 (UmD)	45-6000	M_n_: 3000–23,000	[20]
**Modified prepolymer**	Desmophen 1019–55/DMPA/IPDI/HMDA/DEA/DBTDL/Abex EP-110	Heterogeneous	SC: 25–44 pH: 7.7–8.3 -	40–300 (UmD)	30-5500	-	[39]
**Acetone**	PBA/AAS/IPDI/TMP/HZ/DBTDL	Heterogeneous	SC: 30 - Z_pot_: −25–−48	200–4000 (UmD)	-	-	[40]
**Modified prepolymer**	HTNR/HRSO/TDI/DMPA/DBTDL	Heterogeneous	SC: 20 pH: 7–8 -	64–195 (UmD)	-	-	[41]
**Modified prepolymer**	PPG/PBA/DMPA/IPDI/IPDA/BD/DBTDL	Heterogeneous	SC: 39–55 - -	76.3–921.9 (UmD)	45.2-6000	-	[42]
**Solvent-free**	PEG/PTMG/MDI/SDBS/SDS	Homogeneous	- - -	12,100 (UmD)	-	-	[43]
**Solvent-free**	PTMG (1000–2000 g mol^−1^)/DMPA/IPDI/SAAS/HZ/DMPA	Homogeneous	SC: 30 - -	800–3000 (UmD)	-	M_w_: 52,890–130,800 PI: 1.31–4.02	[44]
**Solvent-free**	CO_2_-polyols (1350-3500 g mol^-1^)/ DMPA/IPDI/HDI/HMDI/EDA	Homogeneous	SC: - - Z_pot_: −27.8	45–70 (UmD)	µPrep: < 20,000–40,000	M_w_: 112,000 PI: 3.42	[45]

- Not reported. Poly(oxytetramethylene)glycol (PTMG); dimethylol propionic acid (DMPA); isophorone diisocyanate (IPDI); 1,4-butanediol (BD); dibutyltin (IV) dilaurate (DBTDL); polypropylene glycol (PPG); polycarbonate of 1,6- hexane diol (PCHDO); poly(1,4-butylene adipate) diol (PBA); diethyleneglycol (DEG); hydrazine hydrate (HZ); linseed oil (LO); hexamethylene diisocyanate (HDI); hydroxyethyl methacrylate (HEMA); neopentyl glycol (NPG); poly(caprolactone) glycol (PCL); adipic acid (AA); 4,4′-diphenylmethane diisocyanate (MDI); ethylene diamine (EDA); 2,6-di-tert butyl-4-methylphenol (BHT); 1-hydroxycyclohexyl phenyl ketone (Irgacure 184); dibutyltin diacetate (DBTDA); sunflower oil (SFO); 1,4-diazobicyclo[2.2.2] octane (DABCO); titanium isopropoxide (Ti (i-Pr)_4_); 4,4′-dicyclohexylmethane diisocyanate (H_12_MDI); ammonium persulfate (APS); isobornyl acrylate (IBOA); 2-(perfluorooctyl)ethyl methacrylate (HDFDMA); butyl acrylate (BA); poly(hexylene adipate-isophthalate) polyester diol (Desmophen 1019-55); 1-butanol (BOL); 1,6-hexamethylene diamine (HMDA); diethyl amine (DEA); ethoxylated nonylphenol ammonium sulfate (Abex EP-110); 2-[(2- aminoethyl)amino]ethanesulfonic acid sodium salt (AAS); trimethylol propane (TMP); hydroxyl telechelic natural rubber (HTNR); hydroxylated rubber seed oil (HRSO); toluene diisocyanate (TDI); isophorone diamine (IPDA); polyethylene glycol (PEG); sodium 2,4-diaminobenzenesulfonate (SDBS); sodium dodecyl sulfate (SDS); sodium 2-[(2-amino ethyl) amino] ethane sulfonate (SAAS); CO_2_-polyols (1350–3500 g mol^−1^).

**Table 2:** Summary of gathered examples of WBPU and WBPUU prepared from conventional and alternative emulsifiers highlighting the chemical composition (WBPU composition), internal emulsifier nature (IEN), internal emulsifier content (IEC), and particle average size (PS).

WBPU Composition	IEN (Designation/Nature)	IEC (% wt)	PS (nm)	Reference
**PPG/TDI/DMPA**	DMPA/anionic emulsifier	4.95	35–225	[61]
**PBA/PMA/PTMG/PPG/IPDI/H_12_MDI/TDI/MDI/BD/TMP/HZ/DMPA**	DMPA/anionic emulsifier	1.6–2.4	100–8000	[62]
**PEG/PTMG/MDI/SDBS**	PEG/nonionic polyol SDBS/anionic chain extender	3.6–4.0 (PEG) 4.3–8.3 (SDBS)	-	[63]
**PBA/DHA/IPDI**	DHA/anionic polyol	31.70	92	[64]
**MPP/Bayhydur^®^ 3100 polyisoc.**	MPP/anionic polyol	0–20	150–320	[65]
**Phospol/IPDI/HDO/APTES**	Phospol/anionic polyol	47–53	32–68	[66]
**PPO/TDI/DMBA/APTES/SDS**	DMBA/anionic emulsifier SDS/external surfactant	3.65 (DMBA) 2.0 (SDS)	189.6–293.4	[67]
**PEG/HDI/LYS**	PEG/nonionic polyol LYS/anionic chain extender	64.1–71.3 (PEG) 10.4–14.0 (LYS)	-	[68]
**MAHCSO/IPDI/HDO/DHZ**	MAHCSO/anionic polyol	57–58.7	41–176	[69]
**MAHCSO/TDI/HDO/PMDA/BPOTCDA/HFIPDA**	MAHCSO/anionic polyol	61–72	23–240	[70]
**PTMG/PEG/MDI/SDBS**	PEG/nonionic polyol SDBS/anionic chain extender	0.38–0.42 (PEG) 3.5–6.8 (SDBS)	-	[71]
**PCL/H_12_MDI/DMPA/BES/BD**	DMPA/anionic emulsifier BES/anionic chain extender	2.1–8.7 (DMPA) 3.7–14.4 (BES)	28–213 (DMPA) 8.3–168 (BES)	[72]
**CE/PTMG/IPDI/DMPA/EDA**	DMPA/anionic emulsifier	5–6	61.5	[73]
**Oxymer M112/SynDD/IPDI/DMPA/HMD**	DMPA/anionic emulsifier	3	81.2–139.2	[74]
**CO/FA/IPDI**	FA/anionic polyol	28.77 (CO) 68.26 (CO free)	35.11 (with CO) 56.11 (without CO)	[75]
**PEG/PTMG/IPDI/GQAS/EGDE**	PTMG/nonionic polyol	-	-	[76]
**PEG/HDI/LYS**	PEG/nonionic polyol LYS/anionic chain extender	63–71 (PEG2000) and 11–15 (LYS)	-	[68]
**PEG/HMDI/DMPA/MDEA/HTO**	PEG/nonionic polyol DMPA/anionic emulsifier MDEA/cationic emulsifier	-	64–198	[77]
**Poly-G 2056/Priplast 3192/Diexter G 4400-57/IPDI/DMPA/DHSA**	DMPA/anionic emulsifier DHSA/anionic polyol	0–4.5 (DMPA) 9.9 (DHA)	500–22,500	[78]
**PCDL/CO/IPDI/DMPA/EDA/BD/THAM**	DMPA/anionic emulsifier	8.4–8.5	50–125	[79]
**GLY-polyols/Voranol 4701/IPDI/DMPA**	DMPA/anionic emulsifier	5–10.8		[80]
**PCD/PBA/IPDI/DMPA/HZ**	DMPA/anionic emulsifier	5	67–84	[51]
**PPG/IPDI/DPSA/BDSA**	DPSA/anionic-nonionic salt BDSA/anionic salt	(R:DPSA/BDSA) R:2/10–8/10 (DPSA + BDSA) = 5%	190–320	[81]
**PBA/DHA/IPDI/MDI/HDI**	DHA/anionic polyol	28–45 DHA	90–125	[37]
**PTMG2000/PTMEG1000/IPDI/DMPA/SAAS/ HZ**	DMPA/anionic emulsifier SAAS/anionic chain extender	0.2–0.4 (DMPA) 0.4–0.6 (SAAS)	800–3000	[44]
**PET/PPG/IPDI/DMPA/BD**	DMPA/anionic emulsifier	5–10	-	[82]
**PPG/IPDI/DMPA/TMPM/BD/EDA/AEAPTMS/APTES**	DMPA/anionic emulsifier	4	-	[83]

Polypropylene glycol (PPG); toluene diisocyanate (TDI); dimethylol propionic acid (DMPA); poly(1,4-butylene adipate) diol (PBA); poly(1,3-butylene adipate) diol (PMA); poly(oxytetramethylene) glycol (PTMG); isophorone diisocyanate (IPDI); 4,4′-dicyclohexylmethane diisocyanate (H_12_MDI); 4,4′-diphenylmethane diisocyanate (MDI); 1,4-butanediol (BD); trimethylolpropane (TMP); hydrazine hydrate (HZ); polyethylene glycol (PEG); sodium 2,4-diaminobenzenesulfonate (SDBS); methoxylated sunflower oil polyol (DHA); maleopimaric acid-based polyester polyol (MPP); hydrophilically modified polyisocyanate (Bayhydur^®^ 3100 polyisoc); phosphorylated polyol (Phospol); 1,6-hexanediol (HDO); 3-aminopropyl-triethoxysilane (APTES); polypropylene oxide (PPO); 2,2-dimethylolbutanoic acid (DMBA); sodium dodecyl sulfate (SDS); 1,6-hexamethylene diisocyanate (HDI); L-lysine (LYS); maleated cottonseed oil-based polyol (MAHCSO); aliphatic dihydrazides (DHZ); pyromellitic dianhydride (PMDA); 3,3′,4,4′-benzophenone-tetracarboxylic dianhydride (BPOTCDA); 4,4′-hexafluoroisopropylidine-diphtalic anhydride (HFIPDA); poly(ε-caprolactone) (PCL 2000 g/mol); N,N-bis(2-hydroxyethyl)-2-amino ethane sulfonic acid sodium salt (BES); cardanol epoxy polyol (CE); ethylene diamine (EDA); polyester diol (Oxymer M112); synthesized diester diol (antimicrobial agent) (SynDD); 1,6-hexamethylene diamine (HMD); castor oil (CO); linssed oil based fatty acid polyol (FA); L-lysine derivative diamine of gemini quaternary ammonium salt (GQAS); ethylene glycol diglycidyl ether (EGDE); N-methyldiethanolamine (MDEA); hydroxylated tung oil (HTO); polyether polyol (Poly-G 2056); dimer acid-based polyester polyol (Priplast 3192); polyester diol polyol (Diexter G 4400-57); palm oil based polyol from oleic acid (DHSA); polycarbonate diol T5652 (PCDL) (2000 g mol^−1^); 2-amino-2-(hydroxymethyl)-1,3-propanediol (THAM); glycerol-based polyols (GLY-polyols); polyether polyol (Voranol 4701); polycarbonate of 1,6-hexanediol (PCD); polyether diols ionic/nonionic sulfonated salt (DPSA); ionic sulfonate salt diols (BDSA); sodium 2-[(2-amino ethyl) amino] ethane sulfonate (SAAS); polyethylene terephthalate (PET); trymethylol propane monooleate (TMPM); 3-(2-aminoethyl)aminopropyl)trimethoxysilane (AEAPTMS).

**Table 3:** WBPU and WBPUU reinforced with renewable entities, their preparation method, and effect on final properties of the derived materials.

Renewable Entities	Preparation Method	Properties of WBPU and WBPUU Derived Materials	Reference
Eucalyptus CNC (0–5 wt.%)	Acid hydrolysis (65 wt.% H_2_SO_4_)	Low Eucalyptus CNC contents considerably increase film’s tensile strength and Young modulus values. The incorporation of Eucalyptus CNC favor the HS-SS microphase separation.	[91]
CNC from microcrystalline cellulose (MCC) (0–100 wt.% of CNC)	Acid hydrolysis (64 wt.% H_2_SO_4_, 45 °C; 45 min)	Chiral nematic structured CNC/WBPU films were prepared with iridescent coloration that varied with composite composition. Films presented rewritable and tunable photonic properties with a fast responsive ability (solvent polarity and humidity).	[105]
Regenerated cellulose nanoparticles (RCN) from MCC (0–5 wt.%)	MCC dissolved in NaOH 7% and urea 12% solution. Addition of deionised water and centrifugation for separating the RCN and ultrasonicated	The addition of RCN increases the storage modulus and improves mechanical and thermal properties, being greater the effect at higher contents. The degradation of the nanocomposite films via enzymatic hydrolysis was improved with RCN addition.	[106]
Cotton cellulose nanofibrils (CNF) (0–20 wt.%)	CNF acid hydrolysis (64% H_2_SO_4_; 45 °C; 90 min), centrifugation and dialysis	Addition of CNF up to 10 wt.% considerably improve nanocomposite film’s tensile strength. The relative humidity of the systems can modify the mechanical properties CNF interact with the SS of the matrix, increasing the T_g_ and decreasing the crystallinity of the SS.	[90]
CNC from sisal fibres (0–10 wt.%)	Fibres mixture with ethanol/toluene solvents mixture for removing extractives. Alkali treatment (7.5 wt.% NaOH; 90 min) for removing hemicellulose and lignin. Acetylation treatment (HNO_3_ + acetic acid; 30 min). Acid hydrolysis (H_2_SO_4_ 64 wt.%, 45 °C, 45 min)	CNC act as nucleating agent of polyurethane SS, and enhance the mechanical properties (Young modulus) of the nanocomposite films, maintaining high elongation values.	[107]
CNC (0–1 wt.%)	MCC acid hydrolysis (64 wt.%; 45 °C; 2 h), centrifugation, dialysis	CNC showed strong interfacial interactions with the WBPU matrix. The nanocomposites were employed as finishing agents in wool fabrics, resulting in greater tensile strength and decreasing area-shrinkage rate (potential in the textile field).	[96]
CNC from Eucalyptus kraft wood pulp (0–1 wt.%)	Acid hydrolysis (64 wt.%; 50 °C; 50 min), centrifugation, dialysis	CNC incorporation route controlled the WBPU-CNC interaction degree, conditioning the phase separation of the segments, morphology, interfacial adhesion, and mechanical properties of the final composite films.	[93]
CNC from MCC (0–3 wt.%)	Acid hydrolysis (64 wt.% H_2_SO_4_; 45 °C; 30 min), centrifugation, dialysis	The different incorporation routes of CNC to the WBPU, lead to different dispositions in the matrix, tailoring thermal, mechanical, and hydrophilic behavior, providing a suitable stress transfer in the nanocomposite films.	[92]
Starch nanocrystals (StNC) from Pea starch (35/65 amylose/amylopectin) (0–30 wt.%)	St acid hydrolysis (3.16 M H_2_SO_4_; 40 °C; 10 days), centrifugation, washed with distilled water	The incorporation of StNC to WBPU led to an increase in the mechanical strength and Young modulus (E) (optimum composition 10 wt.%) due to the effective interface for the stress transfer in the nanocomposite films. At higher StNC contents E value was enhanced although lower strength values were observed due to self-aggregation of StNC.	[100]
StNC from waxy maise St Cellulose whiskers (CW) from cotton linter pulp	St acid hydrolysis (3.16 M H_2_SO_4_; 40 °C; 5 days), centrifugation and freeze-dried Cellulose acid hydrolysis (30 % (*v*/*v*) H_2_SO_4_; 60 °C; 6 h) centrifugation, dialysis and freeze-dried	The combined incorporation of StN and CW to the WBPU originated a synergistic effect in the nanocomposite films. The nanofillers’ different morphology and the strong hydrogen bonding interactions, among them and with the matrix, were reflected in strong networks with enhanced mechanical and thermal properties, compared with the matrix.	[108]
Vinyltrimethoxysilane (VTMS) modified St	Modification of St (HCl at pH 2; 60 °C) hydrolysis of VTMS and condensation between VTMS and St	VTMS modified St was incorporated covalently to the WBPU, enhancing the mechanical behavior and biodegradability of nanocomposite hybrid films in α-amylase solution even comparing with VTMS modified St/WBPU blending systems, due to the effective anchoring of the reinforcement and being those effects more notable in WBPU covalently attached systems.	[98]
High amylose content (80/20 amylose/amylopectin) Corn St (Gelose 80) St/WBPU blends: 90/10; 80/20; 70/30; 60/40; 50/50	Gelatinisation of St by mixing with glycerol (St/gly 80/20 wt.%) in a microwave reactor (140 °C, 15 min, pressure 700–800 kPa)	WBPU and the glycerol plasticized high amylose starch (HAGS) were compatible and influenced by the physical entanglement and hydrogen bonding interactions in the prepared films. The increase of WBPU ratio led to higher flexible and hydrophobic materials. Some compositions presented water repellency, transparency, and mechanical properties similar to LDPE systems, offering great potential as biodegradable packaging materials.	[109]
Corn St (0–30 wt.%) (different incorporation routes)	St gellification (90 °C, 20 min)	The preparation method of WBPU/St dispersions led to chemical and physical interactions, differing from conventional blends and enhancing the films’ degradation ability. Furthermore, the adhesion of microorganisms (B. subtilis) to the surface of the films was enhanced, as well as the susceptibility to alkaline and acid hydrolysis compared with the matrix.	[99]
Cht from crab shell (degree of deacetylation ≥ 75%)	Synthesis of the WBPU chain extended with Cht (in water/acetic acid solution)	WBPU-Cht dispersions were applied in acrylic fabrics by the pad-cure method using the Cht as finishing agents. The fabrics showed improved antibacterial behavior with the incorporation of Cht, being the effect more significant with the increase of Cht molecular weight. The treated acrylic fabrics are suitable for the manufacture of blankets and carpets for hospitals.	[103]
Cht (50,000 g mol^−1^)	Synthesis of the WBPU chain extended with Cht (dissolved in DMSO and BD)	WBPU-Cht dispersions were applied in acrylic fabrics by the pad-dry-cure method. Chitosan-based dispersions improved the tensile strength and crease recovery of the fabrics, also presenting contact-active. The dispersions are presented as multifunctional finishing textile coatings with antibacterial properties	[110]
Cht	WBPU chain extended with Cht (dissolved in 1% of acetic acid)	WBPU-Cht dispersions were applied in cotton/polyester fabrics by the pad-cure method providing remarkable improvement in the antibacterial activity, being presented as antimicrobial finishing coating agents with potential application in polyester/cotton textiles.	[111]
Cht (deacetylation degree > 90%)	Cht and WBPU blends	WBPU-Cht blends were employed for the preparation of hydrogels by macroporous structure on drying. The hydrogels presented improved stability in the aqueous and enzymatic environment, favoring their resistance to biological environments. They also supported adhesion and proliferation of primary dermal rat fibroblast cells and biocompatibility on subcutaneous implantation, being promising materials as wound healing dressings.	[104]
Chitosan from shrimp shells (deacetylation degree ≥ 75%)	Cht hydrophobically modified by isocyanate-terminated polyurethane prepolymers copolymerising them through grafting over the chitosan chain	Preparation of hydrogels and lyophilised hydrogels; both presenting sustained drug release behavior and better biocompatible nature with 3T3 fibroblast cells compared to pure chitosan. The hydrogels exhibited promising potential in drug delivery and tissue engineering applications.	[112]

**Table 4:** Processing technique of WBPU and WBPUU systems and their applicability.

WBPU or WBPUU System	Reinforcement	Processing Technique	Application	Reference
**PUU** **(PCDL/IPDI/MDEA) containing N,N-dihydroxyethyl azobenzene chromophore**	-	Coating onto cotton fabric (coating technique)	Dual-responsive cotton fabric coating (acid condition and UV radiation) for professional garments	[119]
**PU** **(PEG/PPG/TDI/DMPA/EG)**	-	Films by casting	Adhesives on PVC and leather substrates	[120]
**PU or PUU** **(PCL/IPDI/DMPA/MDEA/EDA)**	-	Nanoparticles powder and films by casting	Potential therapeutic application in anti-inflammation and macrophage disorders, and as implanted materials	[121]
**PUU** **(PCL/PEG/IPDI/BD/LYS)**		3-Dimensional porous scaffolds (freeze-drying)	Soft tissue engineering	[113]
**PU** **(PCL/IPDI/DMPA/DB)**	CNC (1, 3 wt.%) and PEO (10 wt.%) as polymer template (then is removed)	Mats by electrospinning	Membranes	[118]
**PU** **(PBA/IPDI/DMPA/BD)**	PEO (15–50 wt.%) as polymer template (then is removed)	Mats by electrospinning	Membranes	[115]
**PUU** **(PCL/PEG/LDI/PD/LYS)**	PVA (blends 0–100 wt.%)	Mats by electrospinning	Biomaterial for tissue engineering	[117]
**Commercial PU** **(Lubrizol Advanced Materials)**	Chitosan (85% DA) (5–15 wt/%)	Mats by electrospinning	Nanofiber filters for air pollution (i.e., air filters and face masks)	[122]
**PUU** **(PVA/PBA/TMXDI/DMPA/EDA)**	PVA (blends 0–100 wt.%)	Mats by electrospinning	Potential application in wound dressing	[116]
**PU** **(PTMG/HMDI)**	Chitosan (DA ≥ 75%)	Lyophilised chitosan-based hydrogels modified with PU (10 and 15% of grafting)	Drug delivery and tissue engineering	[112]
**Commercial PU** **(Sigma-Aldrich)**	Chitosan (DA > 90%) (0–100 wt.%)	Hydrogels scaffolds formed by self-organised in a macroporous structure drying at room temperature	Wound regeneration and healing	[104]
**PUU** **(Polyether polyols/MDI/DMPA/EDA)**	PVA (blend 0–100 wt.%)	Hydrogels by freeze-drying	Potential application in wound dressing in medical devices	[114]
**PU**	Cellulose paper sheet, CdTe nanocrystals quantum dots, carbon dots	Cellulose-based papers (films prepared by dip-coating and casting)	Self-healing luminescent composites for light-emitting materials	[123]
**PU** **(PEG/IPDI/DMPA)**	Chitosan as chain extender (M_n_ 1,000,000 or 150,000 g·mol^−1^)	Immersion of the acrylic fabrics in the PU dispersion	Finishing agent (with antibacterial properties) for acrylic fabrics	[103]
**CO/HMDI/Cellulose acetate**	Cellulose acetate (M_n_ 29,000 g·mol^−1^; 40% acetyl groups)	Modification of cellulose with HMDI and posterior reaction with CO (1:1 wt ratio). Samples were prepared by spreading the adhesive over the substrates	Adhesives for wood, stainless steel, polyethylene, and polyester fabric substrates	[124]
**PUU (PCL/PDLLA/IPDI/DMPA/EDA)**	Forkhead box 3D (Fox3D) (transcription factor and neural crest stem-like cells), and cells	PU hydrogel extruded trough syringe needle 3D bioprinting	Tissue engineering (neuroregeneration or further developed as mini-brain for research and drug screening)	[125]
**PU** **PEDL218/IPDI/DMPA/BD/MDEA**	-	Films by casting	Fibre-reinforced bulletproof composites for ballistic protection applications	[126]
**PU** **PEG/HDI/DMPA/DEG**	10–25 wt.% LiTFSI	Films preparation by casting	All-solid-state lithium-ion batteries	[127]
**PU/Chitosan** **(deacetylation degree 85%)**	AgNPs (0–0.034%)	Membranes by electrospinning	Dental barrier membranes	[128]
**PUU** **(PCL/IPDI/DMPA/EDA)**	-	Films by casting	Paper sizing applications	[129]
**Commercial PU** **(Leasys 5530)**	PU/CNC blends (0–100%)	Films by casting or spread onto a glass slide	Rewritable photonic paper/ink promising in sensors, displays and photonic circuits	[105]
**PU** **(PTMG/HO-PDMS/IPDI/DMPA/BD/HEA/ APS)**	Polydimethylsiloxane (HO-PDMS) (3, 6, 8 and 10 wt.%)	Films by casting	Waterproof coatings	[130]
**PU** **(PEG/IPDI/DMPA/BD)**	Chitosan as a chain extender	Dispersions applied to dyed and printed poly-cotton fabrics by the pad-dry-cure method	Antibacterial textile finishing agent for poly-cotton fabrics	[110]
**PUU** **(PCL/PEG/LDI//PD/LYS)**	-	Light-crosslinking films by casting	Soft tissue engineering scaffolds for tissue repair and wound healing	[2]
**PU** **(PPG/IPDI/BD)**	-	Films by casting	Water-based ink binders	[131]
**PU** **(PEG/IPDI/DMPA)**	Chitosan as a chain extender	Immersion of polyester/cotton fabrics in WBPU and squeezed between two stainless steel rollers	Antibacterial textile finishing agents for polyester/cotton fabrics	[111]
**PU** **(PCL/H_12_MDI/DMBA)**	Acrylate (diacrylate or triacrylate) as photo-curable initiator	3D-digital light processing (DLP) printing	flexible 3D architectures for electronic or soft robots flexible devices	[132]

Polycarbonate diol (PCDL); isophorone diisocyanate (IPDI); n-methyldiethanolamine (MDEA); polyethylene glycol (PEG); polypropylene glycol (PPG); toluene diisocyanate (TDI); dimethylol propionic acid (DMPA); ethylene glycol (EG); poly(caprolactone) glycol (PCL)**;** ethylene diamine (EDA); 1,4-butanediol (BD); L-lysine (LYS); poly(1,4-butylene adipate) diol (PBA); L-lysine ethyl ester diisocyanate (LDI); 1,3-propanediol (PD); poly(vinyl alcohol) (PVA); tetramethylxylene diisocyanate (TMXDI); poly(tetramehylene) glycol (PTMG); 1,6-hexamethylene diisocyanate (HMDI); 4,4′-diphenylmethane diisocyanate (MDI); castor oil (CO); poly (D,L-lactide) diol (PDLLA); polyester diol (PEDL218); hexamethylene diisocyanate (HDI); diethyleneglycol (DEG); polydimethylsiloxane (HO-PDMS); 2-hydroxyethyl acrylate (HEA); ammonium persulfate (APS); poly(caprolactone) glycol (PCL); 4,4′-dicyclohexylmethane diisocyanate (H_12_MDI); 2,2-dimethylolbutanoic acid (DMBA).

**Table 5:** Examples of typical WBPU and WBPUU raw materials restrictions and levels according to the actual European legislation.

Component		Level of Restriction	Regulation/Directive
**Diisocyanate**	4-4′-Dicyclohexylmethane diisocyanate	1 mg/kg in final product expressed as isocyanate moiety	Commission Regulation 1149/2020 [135]
	Isophorone diisocyanate		
	4,4′-Diphenylmethane diisocyanate		
**Polyol**	Polycaprolactone	Without limitations	-
	Polyethylene glycol		
	Polypropylene glycol		
	Polytetramethylene ether glycol		
**Catalyst**	Dibutyltin dilaurate	1 mg/kg in final product expressed as dibutyl	Regulation (EC) No 1907/2006 [133]
	Stannous 2-ethylhexanoate	Without limitations	-
**Internal** **Emulsifier**	Dimethylol propionic acid	SML ^1^ = 0.05 mg/kg	Commission Regulation 10/2011 [139] ^2^
	N-Methyldiethanolamine	SML = 0.05 mg/kg	Commission Regulation 10/2011 [139] ^2^
**Neutralizing Agent**	Triethylamine	3	-
**Chain** **Extenders**	Hydrazine monohydrate	Not allowed	Commission Regulation 1272/2008 [134]
	Diethylenetriamine	SML = 5 mg/kg	Commission Regulation 10/2011 [139] ^2^
	Ethylenediamine	SML = 12 mg/kg ^3^	Commission Regulation 10/2011 [139] ^2^
**Co-Solvent**	Acetone	Without limitations	-
	N-Methyl-2- pyrrolidone	3 mg/Kg in final product	Commission Regulation 588/2018 [137]

^1^ Specific Migration Limit defined according to food contact Regulation (EU) No 10/2011; ^2^ Regulation (EU) No 10/2011 after amended by Commission Regulation (EU) 2020/1245 of 2 September 2020; ^3^ Pre-registered on SVHC List, under evaluation.

## Data Availability

The data presented in this study are available on request from the corresponding author.
